# Renal and Cardiovascular Morbidities Associated with *APOL1* Status among African-American and Non-African-American Children with Focal Segmental Glomerulosclerosis

**DOI:** 10.3389/fped.2016.00122

**Published:** 2016-11-17

**Authors:** Robert P. Woroniecki, Derek K. Ng, Sophie Limou, Cheryl A. Winkler, Kimberly J. Reidy, Mark Mitsnefes, Matthew G. Sampson, Craig S. Wong, Bradley A. Warady, Susan L. Furth, Jeffrey B. Kopp, Frederick J. Kaskel

**Affiliations:** ^1^Stony Brook Children’s Hospital, Stony Brook, NY, USA; ^2^Johns Hopkins Bloomberg School of Public Health, Baltimore, MD, USA; ^3^Basic Research Laboratory, Frederick National Laboratory, NCI, NIH, Leidos Biomedical, Frederick, MD, USA; ^4^Pediatric Nephrology, Children’s Hospital at Montefiore, Bronx, NY, USA; ^5^Division of Nephrology and Hypertension, Cincinnati Children’s Hospital Medical Center, Cincinnati, OH, USA; ^6^Division of Pediatric Nephrology, University of Michigan School of Medicine, Ann Arbor, MI, USA; ^7^Pediatric Nephrology, University of New Mexico, Albuquerque, NM, USA; ^8^Division of Pediatric Nephrology, Children’s Mercy Hospital, Kansas City, MO, USA; ^9^University of Pennsylvania, Philadelphia, PA, USA; ^10^NIDDK, NIH, Bethesda, MD, USA

**Keywords:** cardiovascular, left ventricular hypertrophy, chronic renal disease, FSGS, children

## Abstract

**Background and objectives:**

African-American (AA) children with focal segmental glomerulosclerosis (FSGS) have later onset disease that progresses more rapidly than in non-AA children. It is unclear how *APOL1* genotypes contribute to kidney disease risk, progression, and cardiovascular morbidity in children.

**Design, setting, participants, and measurements:**

We examined the prevalence of *APOL1* genotypes and associated cardiovascular phenotypes among children with FSGS in the Chronic Kidney Disease in Children (CKiD) study; an ongoing multicenter prospective cohort study of children aged 1–16 years with mild to moderate kidney disease.

**Results:**

A total of 140 AA children in the CKiD study were genotyped. High risk (HR) *APOL1* genotypes were present in 24% of AA children (33/140) and were associated with FSGS, *p* < 0.001. FSGS was the most common cause of glomerular disease in children with HR *APOL1* (89%; 25/28). Of 32 AA children with FSGS, 25 (78%) had HR *APOL1*. Compared to children with low risk *APOL1* and FSGS (comprising 36 non-AA and 7 AA), children with HR *APOL1* developed FSGS at a later age, 12.0 (IQR: 9.5, 12.5) vs. 5.5 (2.5, 11.5) years, *p* = 0.004, had a higher prevalence of uncontrolled hypertension (52 vs. 33%, *p* = 0.13), left ventricular hypertrophy (LVH) (53 vs. 12%, *p* < 0.01), C-reactive protein > 3 mg/l (33 vs. 15%, *p* = 0.12), and obesity (48 vs. 19%, *p* = 0.01). There were no differences in glomerular filtration rate, hemoglobin, iPTH, or calcium–phosphate product.

**Conclusion:**

AA children with HR *APOL1* genotype and FSGS have increase prevalence of obesity and LVH despite a later age of FSGS onset, while adjusting for socioeconomic status. Treatment of obesity may be an important component of chronic kidney disease and LVH management in this population.

## Introduction

African-Americans (AA) have higher rates of hypertension (HTN) and kidney disease compared to Americans of European descent (1). In adults of African descent, the presence of high risk (HR) *APOL1* genotype (characterized by the presence of two risk alleles, defined as G1/G1 homozygotes, G2/G2 homozygotes, and G1/G2 compound heterozygotes), preferentially selected by the process of evolution, was found to be associated with non-diabetic or “hypertension-attributed” end-stage renal disease (ESRD), idiopathic focal segmental glomerulosclerosis (FSGS), and HIV-associated nephropathy (2–5). The role of *APOL1* in the adult cardiovascular phenotype is still controversial with some recent findings, suggesting that *APOL1* variants could contribute to atherosclerotic cardiovascular risk, indicating a genetic component to cardiovascular health disparities among adults of African ancestry (6). While studies in adult AA populations demonstrated strong recessive association of *APOL1* G1 and G2 genetic variants with glomerular and vascular disease progression (7), there is limited information on its role in children with chronic kidney disease (CKD), particularly for cardiovascular comorbidities. In NIH FSGS cohort study and in the FSGS-Clinical Trial (FSGS-CT), both of which included children and adults, HR *APOL1* genotype was present in 72% of self-identified AA subjects (8).

Up to 63% of children with early stages of CKD present with arterial HTN (9). In addition to being common, HTN is associated with a greater rate of decline in kidney function and is a known risk of development of ESRD (10). Furthermore, in Chronic Kidney Disease in Children (CKiD), a population with mild to moderate CKD, a high overall prevalence (53%) of systolic HTN was observed (11). Presence of HTN in children with CKD has also been associated with cardiovascular morbidity and development of left ventricular hypertrophy (LVH) (12). LVH is common among hypertensive adults and children with CKD and is more common among AA than in whites (13, 14). While a previous report of two pediatric cohorts representing children with CKD (CKiD) and nephrotic syndrome (Nephrotic Syndrome Study Network; NEPTUNE) showed similar *APOL1* characteristics in terms of FSGS diagnosis and disease progression (15), the association of *APOL1* and cardiovascular comorbidities in the presence of CKD has not been explored. The purpose of this study was to extend previous findings (15), to characterize the distribution of *APOL1* risk alleles in the CKiD cohort and, by comparing children with an underlying FSGS cause of CKD, to describe the prevalence of cardiorenal phenotypes and markers of disease severity associated with the HR *APOL1* genotype.

## Materials and Methods

### Subjects

The study population comprised of children enrolled in the CKiD Study, an ongoing multicenter prospective cohort study of children aged 1–16 years with mild to moderate kidney disease (16).

### Clinical and Laboratory Testing

All clinical and laboratory data in the analysis were based on the first available observation, either at study entry or 6 months after, and were categorized by indicators of renal, cardiovascular, and metabolic health. Echocardiography data were collected 1 year after study entry according to study protocol. Race, sociodemographic, and therapy use were self or parental reported.

#### Genetic Testing

DNA was obtained from National Institute of Diabetes and Digestive and Kidney Disease (NIDDK) genetics repository of immortalized lymphocyte cell lines based on a sample of whole blood taken at the 6-month-old study visit among those who consented to genetic testing. *APOL1* variants G1 (rs73885319, S342G and rs60945101, I384M) and G2 (rs71785313, NY deletion) were genotyped.

The number of kidney risk alleles defined *APOL1* risk groups: low risk (LR) was defined by 0 or 1 risk allele (G0/G0 homozygous, or G1/G0 and G2/G0 heterozygous), and HR was defined by 2 risk alleles (G1/G1 and G2/G2 homozygous, or G1/G2 compound heterozygous), where G0 represents the ancestral alleles at both rs73885319 and rs71785313 sites.

Indicators of renal health included glomerular filtration rate (GFR) measured by plasma disappearance of iohexol or estimated by CKiD-developed equations based on serum creatinine, cystatin C, and BUN, when iohexol GFR was not available. Proteinuria was assessed as first morning urine protein (milligrams)/creatinine (milligrams) ratio (uPCR).

Indicators of cardiovascular health included the average of three in-clinic measurements of blood pressure and left ventricular mass index (LVMI in g/m^2.7^) as measured by echocardiography. Uncontrolled HTN was defined as systolic blood pressure (SBP) or diastolic blood pressure (DBP) ≥95th percentile for age, sex, height according to the fourth report (17). LVH was defined as LVMI ≥ age- and sex-specific 95th percentile based on the normal population (18). All data were measured and defined by the CKiD study protocol, as described previously (11, 16, 19).

Indicators of metabolic health included total, HDL, and LDL cholesterol (milligrams per deciliter), triglycerides (milligrams per deciliter) with age- and sex-defined categorical definitions for abnormally high total cholesterol, low HDL, and high LDL cholesterol, based on the normal population. Hemoglobin (grams per deciliter), calcium (milligrams per deciliter), phosphate (milligrams per deciliter), and calcium × phosphate product were measured. Hemoglobin was categorized by anemia status based on age- and sex-defined levels of the normal population, and phosphate levels were categorized as high based on age-defined levels of the normal population. Intact parathyroid hormone (iPTH) and C-reactive protein (CRP) were also measured (19), with high sensitivity CRP primarily used (*n* = 47) and when missing, informed by wide-range CRP data (*n* = 18; missing both *n* = 3). High CRP was defined as CRP > 3 mg/l.

The CKiD Study protocol was approved by the institutional review boards at the participating institutions, and all subjects gave informed assent or consent. Genetic testing was approved by the NIDDK Institutional Review Board.

### Statistical Analyses

Since 89% of children with the HR *APOL1* profile had FSGS, the appropriate comparison group to determine differences related to *APOL1* were children with LR *APOL1* genotype and FSGS. Fisher’s exact test for categorical variables and the Wilcoxon rank-sum test for continuous variables were used to compare univariate differences by *APOL1* risk status among those with a diagnosis of FSGS.

Since lower socioeconomic status (SES) was associated with AA race in this North American cohort, and low SES is associated with disease progression (20), SES is a potential confounder as the exposure groups largely differ by race. To adjust for confounding due to SES, we used inverse probability of exposure weights (IPWs) (21, 22). The IPWs were generated from a logistic regression model with HR *APOL1* as the outcome and variables related to SES as the predictors: income less than $36,000, maternal education less than college, the presence of any public insurance and missing at least one dose of antihypertensive therapy in the past 7 days (self-reported). The inverse of the predicted probability of observed exposure was stabilized to the marginal probability of each group (for HR *APOL1*: 25/68 = 0.37; and for LR *APOL1*: 43/68 = 0.63). Selected cardiovascular, metabolic, inflammatory, and other risk factors were compared to exposure group in adjusted analyses using weighting by IPWs in logistic regression models to obtain prevalence odds ratio comparing the HR *APOL1* group to the LR *APOL1* group. The outcomes were obesity, high LDL cholesterol, uncontrolled HTN, LVH, and CRP > 3 mg/l. Multiple imputation was used to account for missing data, and the results are presented unadjusted (univariately, with imputation where applicable) and adjusted to account for the effect of confounding by SES.

## Results

**Figure 1:**
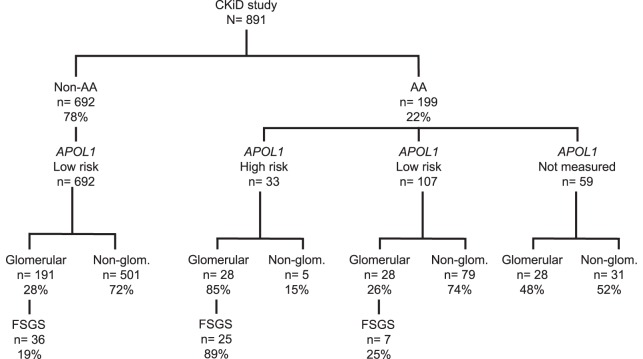
**Distribution of CKiD children by race [African-American (AA) vs. non-African-American (non-AA)], *APOL1* genotypes, and CKD diagnoses [glomerular disease (Glom), non-glomerular disease (Non-Glom), focal segmental glomerulosclerosis (FSGS)] All genotyped non-AA (*N* = 37) were found to have LR *APOL1* status, providing a justification for classifying non-AA children as low risk**.

A total of 891 children were enrolled in CKiD, of whom 199 (22%) were AA; Figure 1 presents the distributions of *APOL1* genotypes and CKD diagnoses by race. Among 140 genotyped AA children, HR *APOL1* status was present in 33 (24%), and among these 33 individuals 28 (85%) had an underlying glomerular cause of CKD, whereas only 5/33 (6%) had a non-glomerular cause of CKD, *p* < 0.001. Of those 28 subjects with HR *APOL1* status and a glomerular disease, 25/28 (89%) had a diagnosis of FSGS. In contrast, among 191 non-AA children with a glomerular CKD cause, only 36 (19%) had an FSGS diagnosis, *p* < 0.001. Of the 28 AA children with LR *APOL1* status (defined as presence of 0 or 1 risk allele), and glomerular CKD, 7 (25%) had a diagnosis of FSGS (difference between HR and LR *APOL1* AA, *p* < 0.001). *APOL1* genotyping was not performed in 59 children: 20 did not consent to genetic testing, 19 dropped out of the study prior to sample collections, 9 were not measured due to missing samples, and 11 samples became available only after genotyping had been completed. Of these 59 children, 47% had a glomerular cause of CKD; 39% of these had a diagnosis of FSGS. The similar proportions of glomerular diagnoses and FSGS among AA LR *APOL1* and non-AA LR *APOL1* children (i.e., 25 and 19%, respectively) suggest potentially similar pathologic mechanisms and were distinct from the distributions observed among AA children with HR *APOL1*. The proportion (47%) of FSGS diagnoses among children with unmeasured *APOL1* status is intermediate between that observed among those with HR (85%) and LR (25%), suggesting that this group with unmeasured genotypes comprises both HR and LR profiles. A total of 37 non-AA children were genotyped, and all were identified as having LR *APOL1* status (36 had 0 risk alleles and 1 harbored 1 risk allele). This provided justification for classifying non-AA children as LR. A full list of diagnoses by race and *APOL1* status is provided in Table S1 in Supplementary Material.

We compared the prevalence of renal and cardiovascular risk factors by *APOL1* status and AA race within FSGS diagnosis group (*n* = 68). Since there were only seven subjects with LR *APOL1* and AA race, we pooled both AA (*n* = 7) and non-AA (*n* = 36) children as the reference population for statistical comparison with AA children with HR *APOL1* and FSGS (*n* = 25). Table 1 presents the clinical, sociodemographic, and renal characteristics among those with FSGS, stratified by race and *APOL1* risk status. There were similar proportions of boys and despite similar ages at study entry, those with HR *APOL1* were significantly taller (*p* = 0.003) and heavier (*p* < 0.001), compared to children with LR *APOL1*. This was reflected in the height and weight percentiles standardized to age and gender (i.e., normal, median height, and weight percentiles are equal to 50): those with HR *APOL1* had a median height and weight percentiles significantly higher as compared to those with LR *APOL1, p* = 0.033 and *p* < 0.001, respectively. Body mass index (BMI) was significantly higher among the HR *APOL1* children (*p* < 0.001), and this group had a much higher proportion of obesity (BMI > 85th percentile adjusted for age and gender; 48 vs. 19%, *p* = 0.01). Consistent with previously published reports (15), children with HR *APOL1* had a higher prevalence of premature birth than children with LR *APOL1* (29 vs. 5%, *p* = 0.011), but this difference was not observed for low birth weight or small for gestational age.

**Table 1:** **Descriptive statistics of sociodemographic, renal health, and therapy use among children with FSGS, by race and *APOL1* risk status (LR, low risk vs. HR, high risk; non-AA, non-African-American; AA, African-American), in the CKiD study (*n* = 68), median (IQR), *n* (%); *p*-values based on Fisher’s exact test or Wilcoxon rank-sum test**.

Variable	Non-AA *APOL1* LR *n* = 36	AA *APOL1* LR *n* = 7	Pooled *APOL1* LR *n* = 43	*APOL1* HR *n* = 25	*p*-Value (pooled LR vs. HR)
Male	21 (58%)	4 (57%)	25 (58%)	11 (44%)	0.318
Black race	0	7 (100%)	7 (16%)	25 (100%)	NA
Age at study entry, years	13.8 [9.6, 15.6]	15.0 [8.2, 16.5]	14.2 [9.5, 15.8]	14.8 [13.0, 15.5]	0.312
Low birth weight (<2500 g)	7 (21%)	1 (17%)	8 (21%)	8 (33%)	0.372
Premature	2 (6%)	0 (0%)	2 (5%)	7 (29%)	0.011
Small for gestational age	7 (23%)	2 (33%)	9 (24%)	7 (30%)	0.765
Low birth weight, premature, or small for gestational age	10 (29%)	2 (29%)	12 (29%)	13 (52%)	0.070
Height, cm	147 [135, 163]	147 [128, 160]	147 [135, 163]	164 [149, 171]	0.003
Height percentile	24 [4, 59]	14 [1, 47]	23 [3, 55]	40 [27, 86]	0.033
Weight, kg	46.4 [34.4, 56.5]	47.1 [27.4, 72.4]	46.4 [34.0, 57.3]	73.5 [60.4, 90.6]	<0.001
Weight percentile	53 [20, 77]	59 [14, 91]	54 [18, 77]	97 [71, 99]	<0.001
Body mass index, kg/m^2^	20.2 [17, 23.9]	19.4 [16.6, 27.1]	20 [17.4, 24.1]	25.1 [22.9, 36.3]	<0.001
BMI percentile	74 [28, 93]	77 [38, 91]	76 [33, 93]	93 [82, 99]	<0.001
Obese	7 (19%)	1 (14%)	8 (19%)	12 (48%)	0.014
**Socioeconomic variables**					
Household income					
<$36,000	15 (43%)	2 (33%)	17 (41%)	12 (48%)	0.409
≥$36,000 and <$75,000	11 (31%)	2 (33%)	13 (32%)	10 (40%)	
≥$75,000	9 (26%)	2 (33%)	11 (27%)	3 (12%)	
Maternal education < college	26 (74%)	6 (86%)	32 (76%)	17 (68%)	0.571
Any public insurance	19 (53%)	2 (29%)	21 (49%)	15 (60%)	0.453
**Renal health**					
Age at CKD onset, years	6.5 [2.5, 11.5]	4.5 [3.5, 3.5]	5.5 [2.5, 11.5]	12.0 [9.5, 12.5]	0.004
Years with CKD	5.3 [3.4, 7.9]	3.7 [2.2, 2.3]	5.2 [3.3, 7.9]	3.3 [1.1, 4.7]	0.008
ieGFR at entry, ml/min/1.73 m^2^	48 [34, 71]	32 [26, 98]	48 [32, 79]	61 [48, 69]	0.132
IeGFR < 45 ml/min/1.73 m^2^	14 (39%)	4 (57%)	18 (42%)	6 (24%)	0.190
ieGFR change per year, %	−7.4% [−3.1%, −2.9%]	−2.2% [−14.0%, −14.2%]	−7.4% [−23.0%, −2.9%]	−8.3% [−14.9%, −1.7%]	0.994
ieGFR change per year, ml/min	−4.1 [−12.3, −1.7]	−2.5 [−7.8, 5.9]	−4.8 [−12.8, −1.7]	−4.4 [−9.6, −1.1]	0.903
uPCR at entry, mg/mg creatinine	1.6 [0.2, 5.5]	1.0 [0.1, 1.2]	1.5 [0.2, 5.5]	0.9 [0.3, 1.8]	0.330
Proteinuria, uPCR > 2	15 (43%)	3 (43%)	18 (43%)	3 (13%)	0.025
**Therapy use**					
Anti-hypertension therapy	31 (86%)	7 (100%)	38 (88%)	25 (100%)	0.150
ACEi/ARB therapy	29 (81%)	7 (100%)	36 (84%)	22 (88%)	0.735
Missed ACEi/ARB in last 30 days	7 (19%)	1 (14%)	8 (19%)	4 (16%)	1.000
Missed ACEi/ARB in last 7 days	6 (17%)	3 (43%)	9 (21%)	10 (40%)	0.103
Steroid therapy	7 (19%)	2 (29%)	9 (21%)	8 (32%)	0.387
Immunosuppression therapy	17 (47%)	5 (71%)	22 (51%)	14 (56%)	0.803

*BMI, body mass index; CKD, chronic kidney disease; ieGFR, measured or estimated glomerular filtration rate; uPCR, urine protein to creatinine ratio*.

To account for the potential confounding effects of SES factors related to race, inverse probability weights were used to adjust for sex, income, maternal education, insurance status, and 7-day adherence to antihypertensive therapy. We found no statistical differences in SES variables between HR and LR *APOL1* genotype groups.

**Figure 2:**
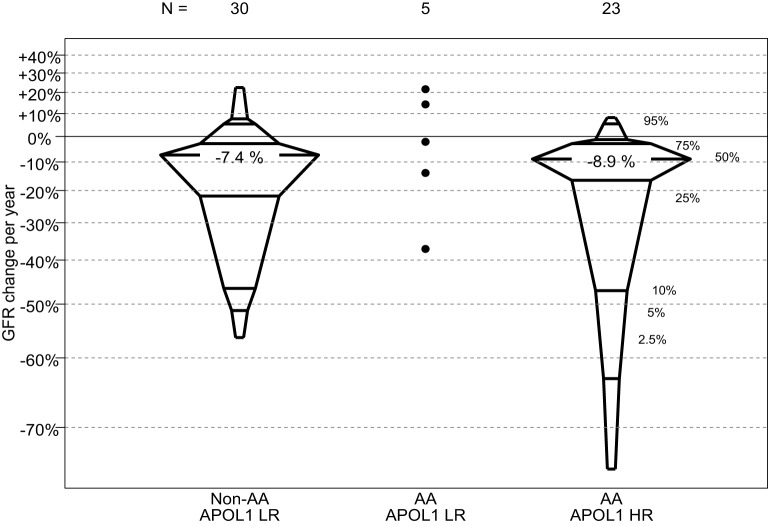
**Percentile boxplots of longitudinal GFR changes based on individual regression equations, expressed as percent change per year, by *APOL1* risk and race**. A total of six non-AA LR participants, two AA LR participants, and two AA HR participants only contributed one GFR measurement and were not included in this analysis.

In this comparison, those children with FSGS and HR *APOL1* had a significantly older age at CKD onset, and significantly shorter duration of CKD than those with LR *APOL1*. Children with HR *APOL1* had a higher GFR at entry although this difference was not statistically significant, *p* = 0.132. Changes in GFR over time were similar to *APOL1* status (Figure 2). Levels of uPCR at entry and over time were also similar, although the HR *APOL1* group had a lower proportion with nephrotic range proteinuria at study entry, *p* = 0.025.

Table 2 and Table S2A,B in Supplementary Material describe characteristics related to cardiovascular health stratified by race and *APOL1* risk status among those with an FSGS cause of CKD. Children with HR *APOL1* had higher SBP (*p* = 0.004), although SBP percentiles (standardized to the normal population for age, gender, and height) were not statistically significant (*p* = 0.132). No significant differences in DBP were observed. The HR *APOL1* children had a higher prevalence of uncontrolled HTN, although it was not statistically significant. Nearly all children, regardless of *APOL1* status received antihypertensive therapy, the HR *APOL1* group had higher (40 vs. 21%) 7-day non-adherence to ACEi/ARB therapy, *p* = 0.103 (Table 1). The proportions receiving glucocorticoid and other immunosuppression therapy were similar by *APOL1* risk status.

**Table 2:** **Cardiovascular and metabolic characteristics among children with FSGS, by race and *APOL1* status**.

Variable	Non-AA *APOL1* LR *n* = 36	AA *APOL1* LR *n* = 7	Pooled *APOL1* LR *n* = 43	*APOL1* HR *n* = 25	*p*-Value (pooled LR vs. HR)
SBP, mmHg	108 [103, 117]	114 [109, 124]	109 [103, 119]	120 [113, 127]	0.004
SBP percentile	62 [43, 81]	88 [48, 97]	64 [45, 88]	79 [55, 95]	0.132
DBP, mmHg	66 [61, 77]	73 [67, 88]	69 [61, 79]	67 [63, 75]	0.889
DBP percentile	64 [36, 88]	90 [58, 99]	68 [37, 92]	57 [46, 85]	0.377
Uncontrolled hypertension	10 (28%)	4 (57%)	14 (33%)	13 (52%)	0.131
LVMI at V2, g/m^2.7^	30.0 [26.9, 33.0]	28.1 [20.7, 28.6]	29.6 [26.9, 33.0]	40.8 [28.1, 52.9]	0.004
LVH at V2	4 (13.8%)	0 (0%)	4 (12%)	9 (45%)	0.003
Total cholesterol, mg/dl	173 [150, 210]	186 [174, 205]	174 [150, 210]	190 [168, 224]	0.222
High total cholesterol	10 (33%)	2 (40%)	12 (34%)	9 (43%)	0.565
HDL cholesterol, mg/dl	53 [41, 61]	53 [50, 66]	53 [41, 63]	51 [41, 60]	0.883
Low HDL cholesterol	4 (13%)	0 (0%)	4 (11%)	0 (0%)	0.286
LDL cholesterol, mg/dl	90 [71, 120]	98 [97, 123]	92 [71, 122]	112 [101, 145]	0.047
High LDL cholesterol	5 (17%)	1 (20%)	6 (17%)	7 (33%)	0.208
Triglycerides, mg/dl	143 [92, 203]	118 [93, 160]	136 [92, 200]	117 [91, 145]	0.441
High triglycerides	18 (60%)	3 (60%)	21 (60%)	10 (48%)	0.578
Hemoglobin, g/dl	12.5 [11.3, 13.4]	11.7 [10.0, 12.9]	12.3 [11.1, 13.3]	12.4 [11.9, 13.6]	0.165
Anemia	17 (47%)	5 (71%)	22 (51%)	8 (32%)	0.139
Calcium, mg/dl	9.2 [8.5, 9.6]	9.6 [8.0, 9.8]	9.2 [8.4, 9.6]	9.4 [9.1, 9.7]	0.179
Phosphate (mg/dl)	4.5 [4.0, 5.1]	4.1 [3.7, 6.0]	4.4 [4.0, 5.1]	4.3 [3.7, 4.7]	0.154
High phosphate	6 (17%)	2 (29%)	8 (19%)	0 (0%)	0.023
Calcium × phosphate	41.7 [37.4, 44.8]	41.0 [36.3, 48.0]	41.4 [37.0, 45.0]	39.9 [34.0, 45.6]	0.504
iPTH, pg/ml	58.0 [35.8, 89.8]	144.3 [40.0, 267.8]	58.0 [38.0, 95.0]	52.0 [39.5, 67.0]	0.399
CRP > 3 mg/l	4 (12%)	2 (29%)	6 (15%)	8 (33%)	0.120

*Median [IQR] or (CI), *n* (%); *p*-values based on Fisher’s exact test or Wilcoxon rank-sum test*.

*SBP, systolic blood pressure; DBP, diastolic blood pressure; LVMI, left ventricular mass index; LVH, left ventricular hypertrophy; V2, visit at 12 months post study enrollment; HDL, high density lipoprotein; LDL, low density lipoprotein; iPTH, intact parathyroid hormone; CRP, C-reactive protein (high sensitivity or wide range)*.

*Uncontrolled hypertension was defined as SBP or DBP ≥ 95th percentile for age, sex, and height, regardless of a self-reported history of hypertension or receiving antihypertensive therapy*.

Increased left ventricular mass index (*p* = 0.004) and a higher prevalence of LVH (*p* = 0.003) were observed in the HR *APOL1* group. LDL cholesterol was higher in the HR *APOL1* group (*p* = 0.047), but no differences in total cholesterol, HDL cholesterol, and triglyceride levels were observed. Higher phosphate was observed among the LR *APOL1* group (*p* = 0.023), but there was no difference in intact parathyroid hormone. Children with HR *APOL1* had a higher proportion of CRP values greater than 3 mg/l (33 vs. 15%, *p* = 0.120).

Table 3 presents odds ratios to describe the associations of the HR *APOL1* profile and selected risk factors and comorbidities. LVH was highly associated with HR *APOL1* (OR: 6.2, 95%CI: 1.6, 24.9), as was obesity (OR: 4.7, 95%CI: 1.5, 14.4). While not statistically significant, HR *APOL1* had a higher relative odds of uncontrolled HTN, elevated CRP, and high LDL cholesterol.

**Table 3:** **Relative odds of selected risk factors and comorbidities comparing AA children with FSGS and HR *APOL1* to pooled children (i.e., non-AA and AA) with FSGS and LR *APOL1* based on logistic regression models**.

	Unadjusted odds ratios (95%CI)	Adjusted odds ratios (95%CI)
Left ventricular hypertrophy	7.97 (1.90, 33.51)	6.22 (1.55, 24.91)
Obesity	4.04 (1.35, 12.11)	4.65 (1.50, 14.43)
Uncontrolled hypertension	2.24 (0.82, 6.17)	2.54 (0.92, 7.00)
C-reactive protein > 3 mg/l	2.74 (0.82, 9.16)	2.41 (0.67, 8.72)
High LDL cholesterol	1.34 (0.44, 4.08)	1.22 (0.40, 3.72)

*Analyses are unadjusted and adjusted for sex, income less than $30,000, maternal education less than college, household having any public insurance, and missing a dose of antihypertensive medication in the past 7 days, as markers of socioeconomic status. Multiple imputation was used to account for missing data*.

*LDL, low density lipoprotein*.

## Discussion

In this cohort of children with FSGS, we demonstrated that AA children with HR *APOL1* genotype were more likely to have FSGS and to have a later age at FSGS onset, compared to AA children with LR genotypes. When restricting analysis to children with FSGS, HR *APOL1* was associated with a greater cardiovascular risk burden, including an increase prevalence of LVH, and obesity, a higher LVMI, BMI, and LDL cholesterol, despite treatment with antihypertensive therapy and adjustment for indicators of SES. Notably, other markers of disease severity were not different between the two groups indicating a potential role for *APOL1* in the etiology of cardiovascular abnormalities.

In children with biopsy confirmed FSGS enrolled in the FSGS-CT, 72% of self-identified AA subjects and 6% of children who identified themselves as non-AA had HR *APOL1* risk alleles (8). In the CKiD population, HR *APOL1* risk genotype was strongly associated with both glomerular disease and FSGS. Strikingly, the prevalence of HR *APOL1* among AA children with FSGS was 76% (25/33), and this was congruent with the 72% observed in the FSGS-CT. The distribution of *APOL1* genotypes observed in the adult normal population did not differ from Hardy–Weinberg expectations for genotype distribution; there was no loss of one particular genotype group or gain in another. This provides population-based statistical evidence that there is no preferential loss of HR genotypes between conception and adulthood (2–5). While our study focused on children who have already been diagnosed with CKD, we are unable to make inferences about the general (i.e., non-diseased) pediatric population. Future studies may wish to characterize the distribution of *APOL1* risk alleles in this group to better understand population risk in children.

Previous studies have established that *APOL1* kidney risk variants are strongly associated with kidney disease among adult AA; specifically, HIV-associated nephropathy (odds ratio, 29; 95% confidence interval, 13–68) and FSGS (odds ratio, 17; 95% confidence interval, 11–26) (2–5). In the CKiD cohort, the HR *APOL1* genotype was associated with the glomerular phenotype in children younger than previously reported; previous studies have established that *APOL1*-associated FSGS is characterized by a tendency to present between ages 15 and 39 years old (7) and in FSGS-CT with median age 17 years (13, 23) (8). The median age of FSGS onset among HR children was 12 years in CKiD, which was older than other LR children with FSGS, a finding previously reported alongside the NEPTUNE children with nephrotic syndrome (15). Additionally, this study found a higher prevalence of prematurity among those with HR *APOL1* compared to AA and non-AA children with FSGS, similar to findings with NEPTUNE that employed a different reference group, suggesting that the prematurity effect extends to non-AA children.

It has been well established that AA children with FSGS have later onset disease than non-AA children and progress more rapidly to ESRD (23, 24). In addition, AA children with nephrotic syndrome have a seven times greater risk of having been diagnosed with FSGS than Hispanics (Mexicans and other immigrants from Central America, but not Puerto Ricans and other Caribbean immigrants) (23), and this has been attributed to genetic factors. The present analysis suggest that among children with CKD, AA children with HR *APOL1* genotype present with later onset kidney disease, and have a higher prevalence of cardiovascular and metabolic comorbidities than patients with LR *APOL1* genotype, despite a shorter duration of disease and the same diagnosis of FSGS. This would suggest that in children with HR genotypes, FSGS might have a different pathophysiology with the added influence of distinctive environmental factors (i.e., obesity and potentially birth history, toxin exposure, and/or additional genetic factors). This in turn may lead to a “vascular/endothelial” phenotype clinically presenting as uncontrollable HTN and LVH leading to “enhanced” podocyte injury; in contrast, non-AA primary FSGS may be characterized by a “primary podocyte” phenotype, earlier onset, and slower CKD progression. It is unclear at present how G1 and G2 alleles may cause endothelial or podocyte damage, although the APOL1 protein forms pores in the lysosomal membrane, leading to pathogen lysis, and a variant APOL1 protein was shown in transgenic mice to induce direct tissue injury (25).

The role of *APOL1* in the adult cardiovascular phenotype remains controversial. Ito et al. (6) using data from the Jackson Heart Study (JHS) and Women’s Health Initiative (WHI) demonstrated that the *APOL1* kidney risk alleles confer a twofold risk of cardiovascular events, without significant changes in left ventricular mass, whereas the Systolic Blood Pressure Intervention Trial (SPRINT) showed the absence of an *APOL1* association with prevalent cardiovascular disease in a non-diabetic adult sample (26). It is possible that cardiovascular disease–*APOL1* interaction may have an age-dependent relationship pattern, similar to one described in early- and late-onset forms of Alzheimer disease associated with apolipoprotein E, epsilon 4 allele, where subjects at both age spectrum (young-old) seem to be affected by the disease penetrance (27). It is unknown if similar age-dependent relationship pattern could also be seen in cardiovascular disease–*APOL1* interaction.

Furth et al. (19) described cardiovascular disease risk factors in the CKiD cohort and found that only 18% of the cohort exhibited CRP values >3 mg/l (independent of GFR). In our study, elevated CRP was not statistically different between HR and LR *APOL1* children with FSGS, suggesting that inflammation is not a driving factor behind LVH. It is possible that differences in LVH are driven by a combination of *APOL1* genotype, underlying metabolic status, obesity/BMI, HTN, and some other unrecognized socioeconomic or racial variables. Our results showed an excess of obesity among those with HR *APOL*1. Since obesity and metabolic dysregulation are risk factors for both cardiovascular disease and CKD onset and progression, managing weight prior to onset in order to prevent or delay disease may be an important clinical consideration. Future studies should seek to clarify whether the effect of *APOL1* on CKD severity is related to obesity or whether these two outcomes are independently influenced by the HR profile. Our finding of increased cardiovascular and metabolic risks associated with HR genotype in children with CKD underscores the importance of early detection and need for more aggressive treatment of obesity, such as early therapy or adherence support, in this population.

It is also possible that the increased cardiovascular risk in the HR children is a consequence of the underlying glomerular disease (FSGS) severity and rate of progression. HTN-misattributed kidney disease in AA has been described in the adult literature (28). Given the cross-sectional nature of this study, future research efforts might investigate a large group of AA children with non-glomerular/congenital kidney disease to investigate the effect of HR *APOL1* on cardiovascular risk, in the absence of podocyte injury and glomerular damage. Since the vast majority of HR children in CKiD had a glomerular disease (85%), we were unable to assess the effect of *APOL1* HR on cardiovascular risk among those with a non-glomerular condition.

This study has several limitations. First, the LR *APOL1* subgroup with FSGS was predominantly non-AA. There were only 7 AA with LR *APOL1* compared to 25 AA children with HR *APOL1* and FSGS, thus highlighting the strong association between HR *APOL1* and FSGS among AA children. Since other forms of glomerular disease among the AA LR *APOL1* children, such as hemolytic uremic syndrome, familial nephritis, or SLE, were less common than FSGS in AA HR group, we chose to restrict our analysis to only those with FSGS to determine the effect of HR *APOL1* among those with the same CKD pathology, which necessitated inclusion of non-AA children with FSGS. Although a minority of non-AA subjects were genotyped; all had LR *APOL1* status, providing a justification for classifying non-AA children as LR. It is possible that some children were misclassified, particularly those of Hispanic ethnicity, although this misclassification would lead to more conservative estimates. Ideally, our study would have included a large group of LR and HR *APOL1* AA children with FSGS. As such, we cannot completely discount genetic (non-*APOL1*) or metabolic factors related to race explaining these effects. A related limitation is the potential confounding effect of race and SES, given the disparate distributions of race between the exposed and unexposed groups. While several key SES variables were not associated with our exposure, 7-day antihypertensive adherence was related to HR *APOL1*. Inverse probability or exposure weights were used to account for potential differences in SES variables in adjusted analyses, but there may be other unmeasured SES variables possibly confounding these relationships. The third limitation was the lack of DNA for analysis in 59 subjects (28 subjects with glomerular disease), reducing statistical power. We did not characterize ancestry-informative markers, and therefore, we did not determine the fraction of African ancestry among subjects, although this information would likely not change any inferences, since the prevalence of HR *APOL1* genotype in this study was nearly identical to that previously reported (5, 7, 8). Finally, this study was limited in sample size and may be considered preliminary data for future research to build upon. These findings need to be replicated in larger populations in both children and adults. Despite these limitations, the association of HR *APOL1* genotype with development of glomerular disease, later onset of FSGS and indications of increased cardiovascular risk among children were compelling.

In conclusion, this study suggests that *APOL1*-associated risks are not restricted to adults only and are present in young children as well. Indeed, in CKiD, cardiovascular abnormalities were more common among AA children with HR *APOL1*. Targeting these established modifiable comorbidities, especially BMI, obesity, and HTN, may be a particularly important component in CKD management to delay ESRD in this HR population.

## Author Contributions

RW developed the idea for this research, participated in analysis and interpretation of the data, and wrote the manuscript. DN participated in the statistical design, analysis and interpretation of the data, and co-wrote the manuscript. SL, CW, JK participated in sample genotyping, analysis, and interpretation of the data, and edited the manuscript. KR and MS participated in analysis and interpretation of the data, and edited the manuscript. BW, SF, MM, CW, and FK participated in the design of the Chronic Kidney Disease in Children prospective cohort study (source of data in this manuscript) and analysis and interpretation of the data, and edited the manuscript.

## Disclaimer

The content of this publication does not necessarily reflect the views or policies of the Department of Health and Human Services, and mention of trade names, commercial products, or organizations does not imply endorsement by the U.S. Government.

## Conflict of Interest Statement

The authors declare that the research was conducted in the absence of any commercial or financial relationships that could be construed as a potential conflict of interest.
