# Supplementary material for: Renal and Cardiovascular Morbidities Associated with APOL1 Status among African-American and Non-African-American Children with Focal Segmental Glomerulosclerosis
Source: Front Pediatr. 2016 Nov 17;4:122. doi: 10.3389/fped.2016.00122 (PMC5110572; doi:10.3389/fped.2016.00122)
Supplement: Supplementary file 1 [file Table_1.DOCX]

Supplementary Table 1. Glomerular disease categories by APOL1 genotype in CKiD Study, % (n).

| Glomerular Disease | Non-AA  Low Risk  (n=191) | AA,  Low Risk  (n=28) | AA,  High Risk  (n=28) |
| --- | --- | --- | --- |
| Focal segmental glomerulosclerosis | 36 (19%) | 7 (25%) | 25 (89%) |
| Hemolytic uremic syndrome | 41 (21%) | 5 (18%) | 0 (0%) |
| Familial nephritis (Alport's) | 12 (6%) | 4 (14%) | 0 (0%) |
| IgA Nephropathy (Berger's) | 15 (8%) | 1 (4%) | 1 (4%) |
| Systemic immunological disease (including SLE) | 29 (15%) | 3 (11%) | 0 (0%) |
| Chronic glomerulonephritis | 18 (9%) | 2 (7%) | 1 (4%) |
| Membranoproliferative glomerulonephritis type I | 12 (6%) | 0 (0%) | 0 (0%) |
| Idiopathic cresentic glomerulonephritis | 6 (3%) | 1 (4%) | 0 (0%) |
| Membranous nephropathy | 4 (2%) | 0 (0%) | 0 (0%) |
| Henoch schonlein nephritis | 7 (4%) | 1 (4%) | 0 (0%) |
| Congenital nephrotic syndrome | 3 (2%) | 0 (0%) | 1 (4%) |
| Membranoproliferative glomerulonephritis type II | 2 (1%) | 1 (4%) | 0 (0%) |
| Sickle cell nephropathy | 0 (0%) | 1 (4%) | 0 (0%) |
| Glomerular Other | 6 (3%) | 2 (7%) | 0 (0%) |

AA- African American, LR- 0, or 1 allele, HR- 2 alleles (G1/G1, or G1/G2, or G2/G2), IgA-Immunoglobulin A nephropathy, SLE- Systemic lupus erythematosus
