# Supplementary material for: Renal and Cardiovascular Morbidities Associated with APOL1 Status among African-American and Non-African-American Children with Focal Segmental Glomerulosclerosis
Source: Front Pediatr. 2016 Nov 17;4:122. doi: 10.3389/fped.2016.00122 (PMC5110572; doi:10.3389/fped.2016.00122)
Supplement: Supplementary file 2 [file Table_2.DOCX]

**Supplementary Table 2a.** Descriptive statistics of sociodemographic, renal health and therapy use among African American children with FSGS, by *APOL1* risk status (LR- low risk vs. HR- high risk, Non-AA- Non-African American, AA- African American), in the CKiD Study (n= 32), Median [IQR], **n (%);** p-values based on Fisher’s exact test or Wilcoxon rank sum test.

| Variable | AA *APOL1* LR  n= 7 | *APOL1* HR  n= 25 | P-value |
| --- | --- | --- | --- |
| Male | 4 (57%) | 11 (44%) | 0.678 |
| Black race | 7 (100%) | 25 (100%) | NA |
| Age at study entry, years | 15.0  [8.2, 16.5] | 14.8  [13.0, 15.5] | 0.982 |
| Low birth weight (<2500g) | 1 (17%) | 8 (33%) | 0.637 |
| Premature | 0 (0%) | 7 (29%) | 0.161 |
| Small for gestational age | 2 (33%) | 7 (30%) | 1.000 |
| Low birth weight, premature or small for gestational age | 2 (29%) | 13 (52%) | 0.402 |
| Height, cm | 147  [128, 160] | 164  [149, 171] | 0.020 |
| Height percentile | 14  [1, 47] | 40  [27, 86] | 0.096 |
| Weight, kg | 47.1  [27.4, 72.4] | 73.5  [60.4, 90.6] | 0.030 |
| Weight percentile | 59  [14, 91] | 97  [71, 99] | 0.021 |
| Body mass index, kg/m^2^ | 19.4  [16.6, 27.1] | 25.1  [22.9, 36.3] | 0.045 |
| BMI percentile | 77  [38, 91] | 93  [82, 99] | 0.065 |
| Obese | 1 (14%) | 12 (48%) | 0.195 |
| Household income |  |  | 0.502 |
| < $36 000 | 2 (33%) | 12 (48%) |  |
| ≥ $36 000 and < $75 000 | 2 (33%) | 10 (40%) |  |
| ≥ $75 000 | 2 (33%) | 3 (12%) |  |
| Maternal education < college | 6 (86%) | 17 (68%) | 0.640 |
| Any public insurance | 2 (29%) | 15 (60%) | 0.209 |
| Age at CKD onset, years | 4.5  [3.5, 3.5] | 12.0  [9.5,2.5] | 0.197 |
| Years with CKD | 3.7  [2.2, 2.3] | 3.3  [1.1, 4.7] | 0.257 |
| ieGFR at entry, ml/min/1.73m2 | 32  [26, 98] | 61  [48, 69] | 0.425 |
| ieGFR  <45 ml/min\|1.73m2 | 4 (57%) | 6 (24%) | 0.165 |
| ieGFR change  per year, % | -2.2%  [-14.0%,- 14.2%] | -8.3%  [-14.9%,-1.7%] | 0.219 |
| ieGFR change  per year, ml/min | -2.5  [-7.8, 5.9] | -4.4  [-9.6, -1.1] | 0.376 |
| uPCR at entry,  (mg/mg creatinine) | 1.0  [0.1, 1.2] | 0.9  [0.3, 1.8] | 0.641 |
| Proteinuria  (uPCR> 2) | 3 (43%) | 3 (13%) | 0.120 |
| Anti-hypertension therapy | 7 (100%) | 25 (100%) | NA |
| ACEi/ARB therapy | 7 (100%) | 22 (88%) | 1.000 |
| Missed ACEi/ARB  in last 30 days | 1 (14%) | 4 (16%) | 1.000 |
| Missed ACEi/ARB  in last 7 days | 3 (43%) | 10 (40%) | 1.000 |
| Steroid therapy | 2 (29%) | 8 (32%) | 1.000 |
| Immunosuppression therapy | 5 (71%) | 14 (56%) | 0.671 |

BMI-body mass index, CKD-chronic kidney disease, ieGFR-measured or estimated glomerular filtration rate, uPCR-urine protein to creatinine ratio

**Supplementary Table 2b.** Cardiovascular and metabolic characteristics among African American children with FSGS, by *APOL1* status. Median [IQR] or (CI), n (%); p-values based on Fisher’s exact test or Wilcoxon rank sum test.

| Variable | AA *APOL1* LR  n= 7 | *APOL1* HR  n= 25 | P-value |
| --- | --- | --- | --- |
| SBP, mmHg | 114  [109, 124] | 120  [113, 127] | 0.294 |
| SBP percentile | 88  [48, 97] | 79  [55, 95] | 0.837 |
| DBP, mmHg | 73  [67, 88] | 67  [63, 75] | 0.132 |
| DBP percentile | 90  [58, 99] | 57  [46, 85] | 0.021 |
| Uncontrolled hypertension | 4 (57%) | 13 (52%) | 1.000 |
| LVMI at V2, g/m^2.7^ | 28.1  [20.7, 28.6] | 40.8  [28.1, 52.9] | 0.042 |
| LVH at V2 | 0 (0%) | 9 (45%) | 0.123 |
| Total cholesterol, mg/dL | 186  [174, 205] | 190  [168, 224] | 0.974 |
| High total cholesterol | 2 (40%) | 9 (43%) | 1.000 |
| HDL cholesterol, mg/dL | 53  [50, 66] | 51  [41, 60] | 0.696 |
| Low HDL cholesterol | 0 (0%) | 0 (0%) | NA |
| LDL cholesterol, mg/dL | 98  [97, 123] | 112  [101, 145] | 0.649 |
| High LDL cholesterol | 1 (20%) | 7 (33%) | 1.000 |
| Triglycerides, mg/dL | 118  [93, 160] | 117  [91, 145] | 0.820 |
| High triglycerides | 3 (60%) | 10 (48%) | 1.000 |
| Hemoglobin, g/dL | 11.7  [10.0, 12.9] | 12.4  [11.9, 13.6] | 0.047 |
| Anemia | 5 (71%) | 8 (32%) | 0.091 |
| Calcium, mg/dl | 9.6  [8.0, 9.8] | 9.4  [9.1, 9.7] | 0.819 |
| Phosphate (mg/dl) | 4.1  [3.7, 6.0] | 4.3  [3.7, 4.7] | 0.615 |
| High phosphate | 2 (29%) | 0 (0%) | 0.042 |
| Calcium x phosphate | 41.0  [36.3, 48.0] | 39.9  [34.0, 45.6] | 0.523 |
| iPTH, pg/mL | 144.3  [40.0, 267.8] | 52.0  [39.5, 67.0] | 0.180 |
| CRP > 3 mg/L | 2 (29%) | 8 (33%) | 1.000 |

SBP- systolic blood pressure, DBP- diastolic blood pressure, LVMI- left ventricular mass index, LVH- left ventricular hypertrophy, V2- visit at 12 months post study enrollment, HDL- high density lipoprotein, LDL- low density lipoprotein, iPTH- intact parathyroid hormone, CRP- C-reactive protein (high sensitivity or wide range). Uncontrolled hypertension was defined as SBP or DBP ≥ 95^th^ percentile for age, sex and height, regardless of a self-reported history of hypertension or receiving antihypertensive therapy.
